# Periumbilical laparoscopic surgery through triple channels using common instrumentation

**DOI:** 10.3892/etm.2013.932

**Published:** 2013-01-29

**Authors:** JIA-YUN GE, LING WANG, HAO ZOU, XIAO-WEN ZHANG

**Affiliations:** Department of Hepatobiliary and Pancreatic Surgery, Second Affiliated Hospital, Kunming Medical University, Kunming 650101, P.R. China

**Keywords:** laparoscopic cholecystectomy, periumbilical triple channels, common instruments

## Abstract

Single-port laparoscopic technology is effective in minimally invasive surgery. However, this technology requires expensive instrumentation. In the present study, an alternative minimally invasive technique, periumbilical laparoscopic surgery through triple channels using common instrumentation, is introduced. Increased use of this new technique may be worthwhile since its results are comparable with those of single-port laparoscopic cholecystectomy. Periumbilical laparoscopic cholecystectomy using common instruments through triple channels was performed in 78 cases of simple cholecystolithiasis and 30 of gallbladder polyposis which were compared with a control group consisting of 356 cases of traditional laparoscopic cholecystectomy. The surgery was successfully completed using common instrumentation without complications in 106 cases from the experimental group. However, in 2 cases the surgery was changed to the traditional laparoscopic cholecystectomy due to bleeding in the area of Calot’s Triangle. No statistical differences in the amount of surgical bleeding, intestinal function restoration time, hospitalization time and cost were observed between the two groups. The mean surgery times of the experimental and control groups were 110.31±14.57 and 43.98±7.64 min, respectively. The difference in surgery times was statistically significant. Based on relevant experience of the process of laparoscopic cholecystectomy, the periumbilical triple channel technique is safe and feasible for use in basic-level medical units and does not produce abdominal scarring so an unblemished appearance is preserved. Moreover, this approach only requires common laparoscopic instruments.

## Introduction

The first laparoscopic cholecystectomy was performed by Mouret (1) in 1987. Since then, the laparoscopic technique has been used widely in every type of common surgery. While the majority of surgeons have focused on reducing the number and size of surgical incisions, other surgeons are taking a more challenging surgical approach, characterized by making the incisions more covert to cause fewer scars. This advanced technology, which was put forward by Navarra *et al* in 1992 (2), is termed laparoendoscopic single-site surgery (LESS). A different surgical approach using a natural orifice in the body such as the gastrointestinal tract or vagina, known as natural orifice transluminal endoscopic surgery (NOTES), was reported by Marescaux *et al* (3) in 2007. NOTES is closer to the concept of minimally invasive surgery (4–9). In China, NOTES has been performed by numerous medical centers. However, NOTES requires higher surgical skill and expensive devices and may also result in intra-abdominal infection and puncture mouth leakage (10–12), which have limited its use in China. In actual clinical work, LESS is easier to popularize due to the lower costs and surgical skill required. A number of medical centers have implemented modified LESS. A number of medical centers have implemented modified LESS techniques, including transumbilical endoscopic surgery and single-port LESS (13,14). These techniques are locally implemented improvements that have been made to the traditional laparoscopic technology.

Single-port laparoscopic technology is widely used in minimally invasive surgery. Due to the expensive instrumentation and (15,16) higher surgical skills that are required, as well as the intra-abdominal infection and puncture mouth leakage produced, the promotion of its use is challenging. For this reason, a minimally invasive technique, periumbilical laparoscopic surgery through triple channels using common instrumentation, is introduced in the present study. Our periumbilical laparoscopic surgery through triple channels has been demonstrated to be a safe and effective laparoscopic procedure, with successful experiences in 106 cases of laparoscopic cholecystectomy. These results motivated us to perform the present study. The results achieved by the technique are comparable with those of single-port laparoscopic cholecystectomy.

## Materials and methods

### Ethics

The study was conducted in accordance with the declaration of Helsinki and with approval from the Ethics Committee of the Second Affiliated Hospital of Kunming Medical University. Written informed consent was obtained from all participants.

### General information

Based on the reports from B-ultrasound, 108 cases of completed periumbilical laparoscopic cholecystectomy (78 cases of simple cholecystolithiasis and 30 cases of gallbladder polyposis) treated using common instrumentation through triple channels were selected for the present study. Comparative analysis was performed with a control group consisting of 356 patients who had undergone traditional laparoscopic cholecystectomy between June 2009 and May 2011. In the experimental group, the youngest patient was 32 years old and the oldest was 66 years old. The mean age was 52.96±6.72 years old. In the control group, the youngest patient was 28 years old and the oldest was 76 years old. The mean age was 54.87±7.81 years old. The tests and preparations for all patients prior to surgery were the same. No statistically significant differences were observed between the two groups with regard to age, gender and type of disease (Table I).

### Surgical methods

**Figure 1:**
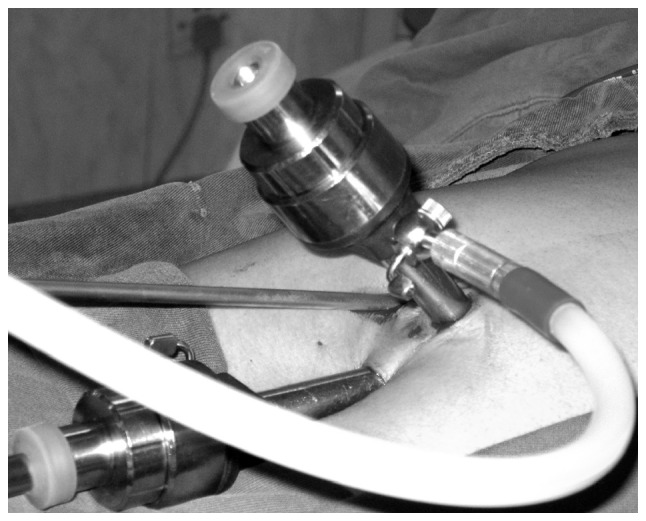
Photograph of the trocar used.

Conventional laparoscopic cholecystectomy was used for the control group and periumbilical laparoscopic cholecystectomy through triple channels was performed for the experimental group. In the experimental group, we used common instrumentation and a method that reduced the interference between troca and air leakage. In addition, this method does not require the operator to wipe the lens repeatedly (Fig. 1).

### Surgical position

The surgical positions of the 2 groups were the same, wherein the patients were turned left 30° and the head was turned 25° upwards. However, for the experimental group, the main surgeon stood behind the assistant, as it was more convenient.

### Surgical instruments

The two techniques used the same surgical equipment, including a laparoscope (model, Xenon Nova 300; Code, 20134020; KARL STORZ, Tuttlingen, Germany), gallbladder grasper, separating pliers, dissecting scissors, electrical separating hook, puncture needle, suction tube, gasless machine and television pickup system. No special laparoscopic instruments were employed.

### Experimental method

Experimental data, including the amount of surgical bleeding, surgery time, intestinal function recovery time, hospitalization time and hospitalization cost were analyzed using statistical tests (Table II).

### Statistical analysis

A Student’s t-test was used to compare differences between the groups using SPSS 10.0 software (SPSS Inc., Chicago, IL, USA). P<0.000312 was considered to indicate a statistically significant result.

## Results

**Figure 2:**
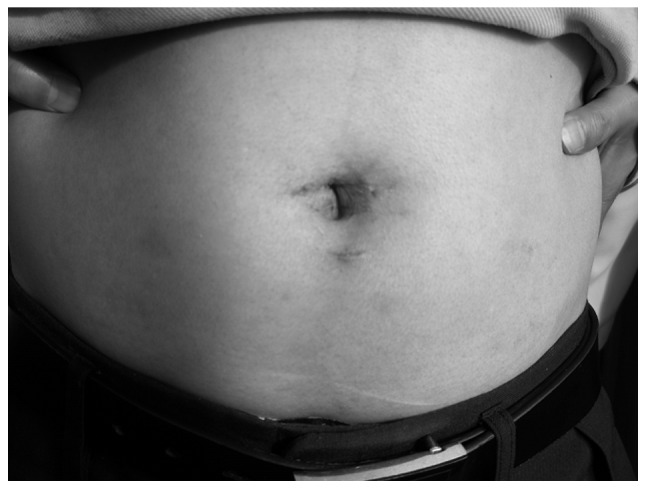
Photograph of a patient one month after surgery.

Surgery was successfully completed in the 106 cases from the experimental group using common instrumentation without complications. However, in 2 cases a change to the traditional laparoscopic cholecystectomy approach was required due to insufficient gallbladder artery clipping. No statistically significant differences in the amount of surgical bleeding, intestinal function recovery time, hospitalization time and hospitalization cost were observed between the groups. However, a statistically significant difference was observed in the surgery time (Fig. 2).

Out of the 106 cases in the experimental group, 84 were followed up for 1 to 28 months, with a mean of 17.4 months. Of the cases followed up, 79 showed no specific discomfort, while 3 experienced abdominal pain without jaundice at 3 to 6 months after surgery. The pain was diagnosed as gallbladder stump inflammation through magnetic resonance cholangiopancreatography (MRCP) tests. The symptoms were relieved following anti-inflammatory allopathic therapy and the patients subsequently reported no discomfort. A further 2 cases experienced unbearable abdominal pain without jaundice at 9 to 13 months after surgery. The pain was diagnosed as residual stones. The residual stones of the 2 patients were removed using endoscopic sphincterotomy (EST) and followed up.

## Discussion

With the rapid development of minimally invasive techniques, the ambitions of numerous surgeons have become achievable. LESS and NOTES are typical examples of such techniques. A considerable number of studies have been performed (17–19) and advancements have been achieved. In our case, however, problems arose during the process of popularization mainly due to the disadvantages, including the use of specialized instrumentation and higher surgical costs. A number of medical centers (20,21) have used special instruments for periumbilical laparoscopic cholecystectomy and attained positive results. However, popularization of the method is problematic due to regional economic differences and non-conformity of health care policy. Consideration of the reported experiences of these medical centers and our own experience suggests the following advantages for our particular laparoscopic cholecystectomy technique.

Firstly, only common laparoscopic instruments are required to perform laparoscopic cholecystectomy. Despite the cost-saving measures, no abdominal scarring is produced. Surgeons with relevant experience of laparoscopic cholecystectomy may perform this surgery, even if they belong to a basic-level medical unit.

Secondly, the problem of interference between the trocar and air leakage was considerably reduced due to the use of 3 channels. The ‘big triangle’ from the traditional laparoscopic cholecystectomy was replaced by a small triangle around the umbilical opening. In cases of uncontrolled bleeding during surgery, one or two punctures may be established below the xiphoid process or rib bow to stop the bleeding. This was carried out in 2 cases from the experimental group.

Thirdly, this technique is more suitable for basic-level medical units. The technique may be performed as long as a laparoscopic device and a surgeon with experience of laparoscopic cholecystectomy are available in the hospital. This approach involves an improvement in the laparoscopic technique only, so the use of large medical equipment, particularly MRI or special instrumentation, such as flexional separating pliers, is unnecessary and the approach is suitable for use in basic-level medical units. Ultimately, this technique is likely to be be beneficial in less economically developed regions.

However, certain concerns require consideration. These include the full exposure of the Calot’s triangle to prevent bile duct injury and bleeding. Damage due to the electrical conductivity of instruments in a narrow space is another concern. Moreover, suturing the periumbilical incision to prevent umbilical herniation is necessary. Therefore, anesthetic drugs, such as 2% lidocaine, are highly recommended prior to suturing to reduce postoperative analgesic dosage. Absorbable suture materials and careful suturing are also important for cosmesis. As surgical experience increases, the surgery time of this technique may be reduced. At present, it takes 45 to 80 min to perform each operation after experiencing 30 cases. According to the literature, this technique has already been utilized in appendectomy, sigmoidectomy and liver cyst decortication. Furthermore, 12 cases of acute appendicitis have been treated using this technique and satisfactory results were obtained. Lastly, the management of complications is similar to that of the traditional laparoscopic technique.

The current results indicate that the technique is safe and feasible for use in laparoscopic cholecystectomy. It does not produce abdominal scars and preserves an unblemished appearance. Furthermore, the technique is particularly suitable for use in China and only requires common laparoscopic instrumentation. Therefore, popularization of the technique should be implemented.

## Figures and Tables

**Table I:** Patient characteristics.

Factor	Experimental group	Control group	t-value (P-value)	χ^2^-value (P-value)
Age	52.96±6.72	54.87±7.81	−1.689 (0.092)	-
Gender (male/female)	44/64	137/219	-	1.545 (0.214)
Type of disease (stone/polyp)	78/30	287/69	-	0.069 (0.793)

**Table II:** Experimental data.

Factor	Experimental group	Control group	t-value	P-value
Amount of bleeding (ml)	67.81±9.03	65.58±9.15	1.648	0.100
Surgery time (min)	110.31±14.57	43.98±7.64	46.673	0.000
Recovery time of intestinal function (h)	28.88±5.69	30.37±6.78	−1.529	0.128
Hospitalization time (days)	4.00±0.94	3.79±0.66	−1.911	0.057
Hospitalization cost (thousand yuan)	7.4±0.6	7.6±0.7	−1.867	0.063
